# Acute Compartment Syndrome with Multiple Metacarpal Fractures in the Hand of a 5-Year-Old Boy: A Case Report of Successful Resolution with Delayed Primary Closure

**DOI:** 10.3390/jcm14041304

**Published:** 2025-02-16

**Authors:** Gonzalo Uquilla-Loaiza, Fanny K. Tupiza-Luna, Galo S. Guerrero-Castillo, Juan S. Izquierdo-Condoy

**Affiliations:** 1 Departamento de Ortopedia y Traumatología, Hospital Vozandes Quito, Quito 170521, Ecuador; 2 Departamento de Ortopedia y Traumatología, Clínica TOA, Quito 170303, Ecuador; 3 One Health Research Group, Universidad de las Américas, Quito 170124, Ecuador

**Keywords:** acute compartment syndrome, fasciotomy, metacarpal fracture, interstitial pressure, pediatric patient

## Abstract

**Background**: acute compartment syndrome (ACS) is an orthopedic emergency characterized by pathological pressure elevation within a confined anatomical space, posing a significant challenge in pediatric patients. ACS affects children in a very limited proportion, particularly involving the upper extremities, and diagnosis is complicated by subtle manifestations compared to adults. **Case Presentation**: we report on the case of a 5-year-old boy who sustained a crush injury to his right hand, resulting in multiple metacarpal fractures and subsequent ACS. The patient presented with severe pain, hand deformity, and restricted mobility. Radiological evaluation confirmed displaced fractures of multiple metacarpals. Emergency surgical intervention involved six incisions to decompress the dorsal and palmar compartments, release muscles, and decompress the median nerve. Healing involved delayed primary closure monitored every 72 h, resulting in an optimal outcome without complications. **Conclusions**: pediatric upper extremity ACS is rare, complicating diagnosis and treatment due to limited guidelines. This case underscores the effectiveness of early surgical decompression and delayed primary closure in achieving favorable outcomes. Individualized treatment strategies tailored to anatomical considerations and ACS severity are crucial to optimize patient care in pediatric surgical settings.

## 1. Introduction

Acute compartment syndrome (ACS) is an orthopedic emergency characterized by pathological pressure elevation within a confined anatomical space, presenting a significant clinical challenge, especially in pediatric patients [1,2]. This condition occurs when the interstitial tissue pressure exceeds the capillary perfusion pressure, resulting in compromised blood flow and potential ischemic damage [3]. Immediate intervention is crucial to mitigate the risk of irreversible tissue necrosis [4,5]. ACS is more commonly observed in males and primarily affects the lower extremities [6], even in children [1,7].

Although ACS is well-documented in orthopedic literature, its occurrence in children, particularly involving hands, remains relatively rare and is reported in only a limited number of cases [7,8,9,10,11]. Diagnosing and managing ACS in pediatric patients present unique challenges, as clinical presentations often differ from those seen in adults. While ACS is traditionally associated with traumatic injuries such as fractures, crush injuries, and vascular compromise, its subtle manifestations in children can complicate early detection [11,12].

Unlike adults, who often display the classic “5 P” symptoms (pain, pallor, paresthesia, paralysis, and pulselessness), pediatric patients may present with atypical signs such as anxiety, agitation, and increased analgesic requirements, all of which may serve as early indicators of impending ACS [9,13,14]. Although compartment pressure measurement using a blood pressure cuff can be a useful diagnostic tool, its utility is limited compared to clinical assessments. A compartment pressure value of less than 30 mmHg is commonly used as a diagnostic threshold; however, clinical judgment remains the cornerstone for diagnosing ACS, particularly in children [15].

Treatment of ACS typically involves prompt surgical intervention with fasciotomy to alleviate compartmental pressure and restore tissue perfusion [4]. However, optimal strategies for wound closure following fasciotomy in pediatric ACS have not been definitively established [6,16]. Significant variability exists in clinical practices among professionals. Insights from adult literature suggest an initial closure with early consideration of skin grafting to minimize additional procedures and hospital stays associated with delayed primary closure [17]. However, these recommendations have yet to be validated in pediatric settings, where factors such as age-related differences in healing further complicate management decisions and contribute to the lack of consensus on optimal treatment approaches [18,19].

In this context, we present the case of a 5-year-old boy with acute compartment syndrome in the right hand who underwent successful recovery following management with fasciotomy and delayed primary closure.

## 2. Case Presentation

**Figure 1:**
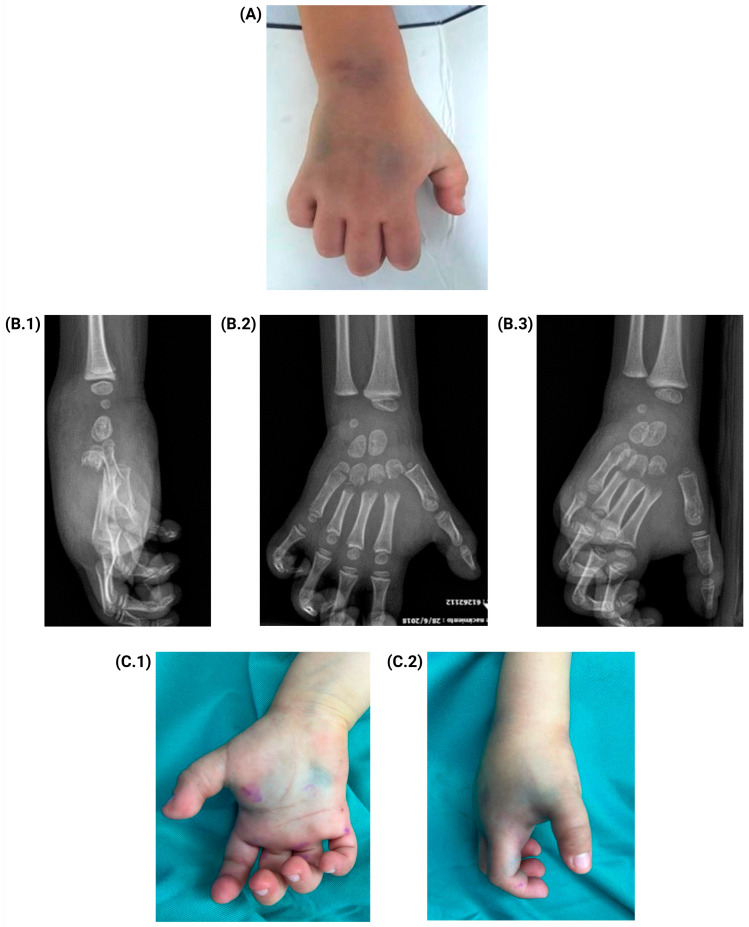
Condition of the patient’s hand on arrival. (**A**) Physical examination of the hand upon arrival at the emergency room. (**B.1**–**B.3**) Lateral, anteroposterior, and posteroanterior radiographs of the hand. (**C.1**,**C.2**) Condition of the patient’s hand prior to resolutive surgery.

A five-year-old male patient with no significant medical or surgical history suffered a crush injury to his right hand from a soccer goal (approximately 60 kg). He presented to the emergency department 30 min after the incident, complaining of severe pain and deformity. On arrival, the physical examination revealed agitation, irritability, marked swelling, and deformity of the right hand, with restricted mobility due to pain and capillary refill of less than two seconds (Figure 1A).

Radiographic examination showed fractures of the second, third, fourth, and fifth metacarpals of the right hand with volar displacement. During the assessment period, the patient became irritable and experienced worsened pain with minimal finger movement (Figure 1B.1–B.3). One hour after arrival, pallor and a decrease in temperature compared to the contralateral limb were noted (Figure 1C.1,C.2), prompting the initiation of a safe surgery protocol.

The procedure involved the compartmental release of the hand through six incisions, carefully placed to ensure adequate decompression. The first incision was made between the second and third metacarpals, while the second was positioned between the fourth and fifth metacarpals. Additional incisions were required in the thenar and hypothenar eminences, as well as in the central palm. A final dorsal longitudinal incision at the level of the first intermetacarpal space was performed when capillary refill in the thumb exceeded two seconds, leading to improved blood perfusion after the release.

**Figure 2:**
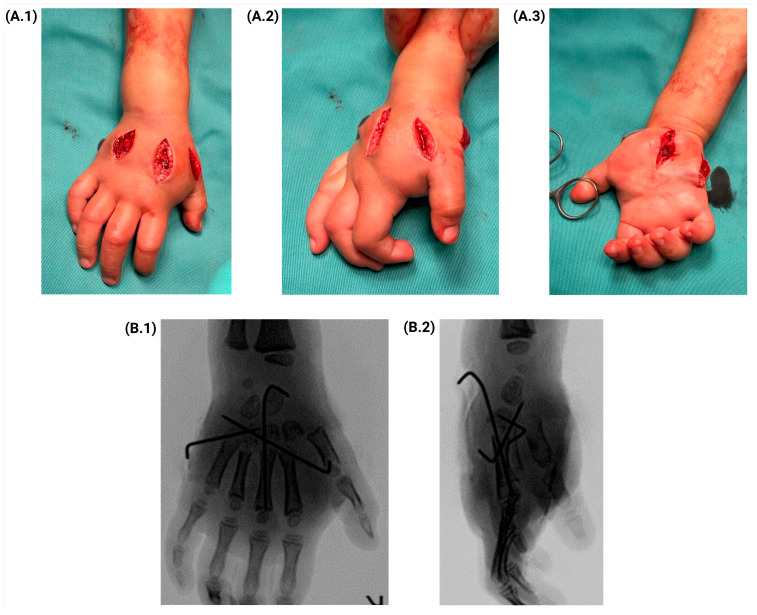
Surgical resolution of the hand. (**A.1**–**A.3**) Illustrations of the incisions to release the hand compartments. (**B.1**,**B.2**) Anteroposterior and lateral radiographs of the post-surgical hand, identifying reduction and Kirschner pins.

Dorsal incisions allowed for decompressive fasciotomy of the interosseous compartments, facilitating the release of the intrinsic and lumbrical muscles. Meanwhile, the palmar incisions enabled the decompression of the thenar and hypothenar muscles. A central incision was essential for relieving the pressure on the median nerve, achieved through the transection of the transverse ligament, along with decompression of the central compartment (Figure 2A.1–A.3). During surgery, an intense infiltration of a subcutaneous hematoma was observed and subsequently drained. Additionally, a closed reduction of a complex carpometacarpal dislocation was performed using percutaneous Kirschner wires under image intensification (Figure 2B.1,B.2).

Surgical closure involved covering the wounds with Vaseline gauze over the incisions and sterile dressings, secured with non-compressive bandages and a palmar cast splint.

Postoperatively, immediate recovery included the restoration of color, temperature, and capillary refill in less than two seconds. Nine hours after surgery, finger mobility was observed with slight pain upon movement and adequate distal sensitivity. The patient was administered cefadroxil and discharged with instructions for continuous monitoring. Physical therapy commenced 48 h after the procedure, and scheduled surgical debridements were performed every 72 h.

**Figure 3:**
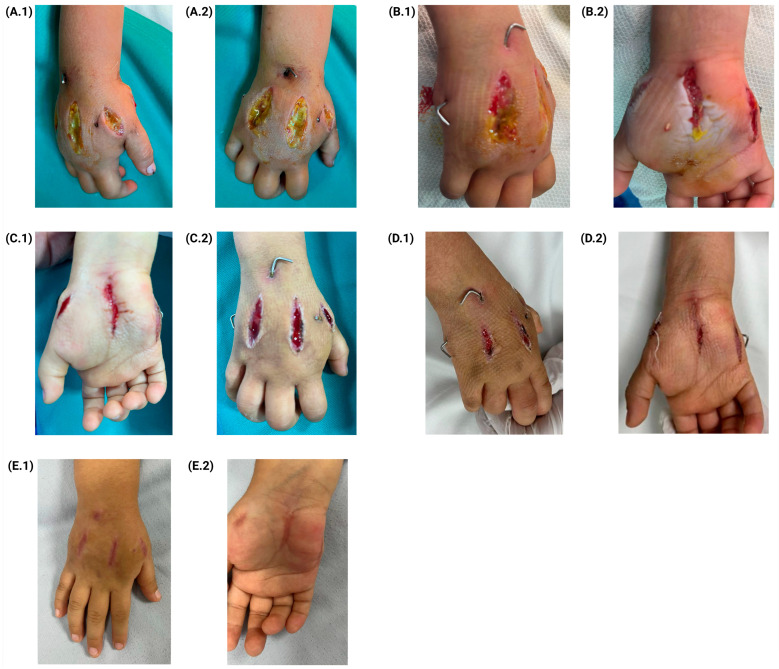
Graphical timeline of patient management and recovery. (**A.1**,**A.2**) Fasciotomy wounds during the first surgical cleaning, 3 days after medical discharge. (**B.1**) Granulation tissue surrounding the thenar eminence, along with evidence of fasciotomy. (**B.2**) Fasciotomy wounds observed during the second surgical debridement, six days post-discharge. (**C.1**,**C.2**) Fasciotomy wounds during the third surgical cleaning, 9 days after medical discharge. (**D.1**,**D.2**) Fasciotomy wounds during the fourth surgical cleaning, 12 days after medical discharge. (**E.1**,**E.2**) Status of the hand after the removal of the Kirschner pins, 21 days after medical discharge.

The patient’s progress showed a favorable improvement. During the first surgical debridement, the open wounds were in good condition, with reduced swelling and capillary refill remaining under two seconds, necessitating surgical irrigation and splinting. By the second debridement, fasciotomies exhibited granulation tissue, the Kirschner wire entry sites showed no signs of infection, and the edema had decreased, with improved approximation of fasciotomy edges. The third debridement revealed a continued wound healing with an adequate pain response, requiring no analgesics. By the fourth and final surgical debridement, wound edges had approximated significantly, indicating sufficient closure. After three weeks, the Kirschner wires were removed under sedation, and the patient had regained mobility (Figure 3A.1–E.2).

**Figure 4:**
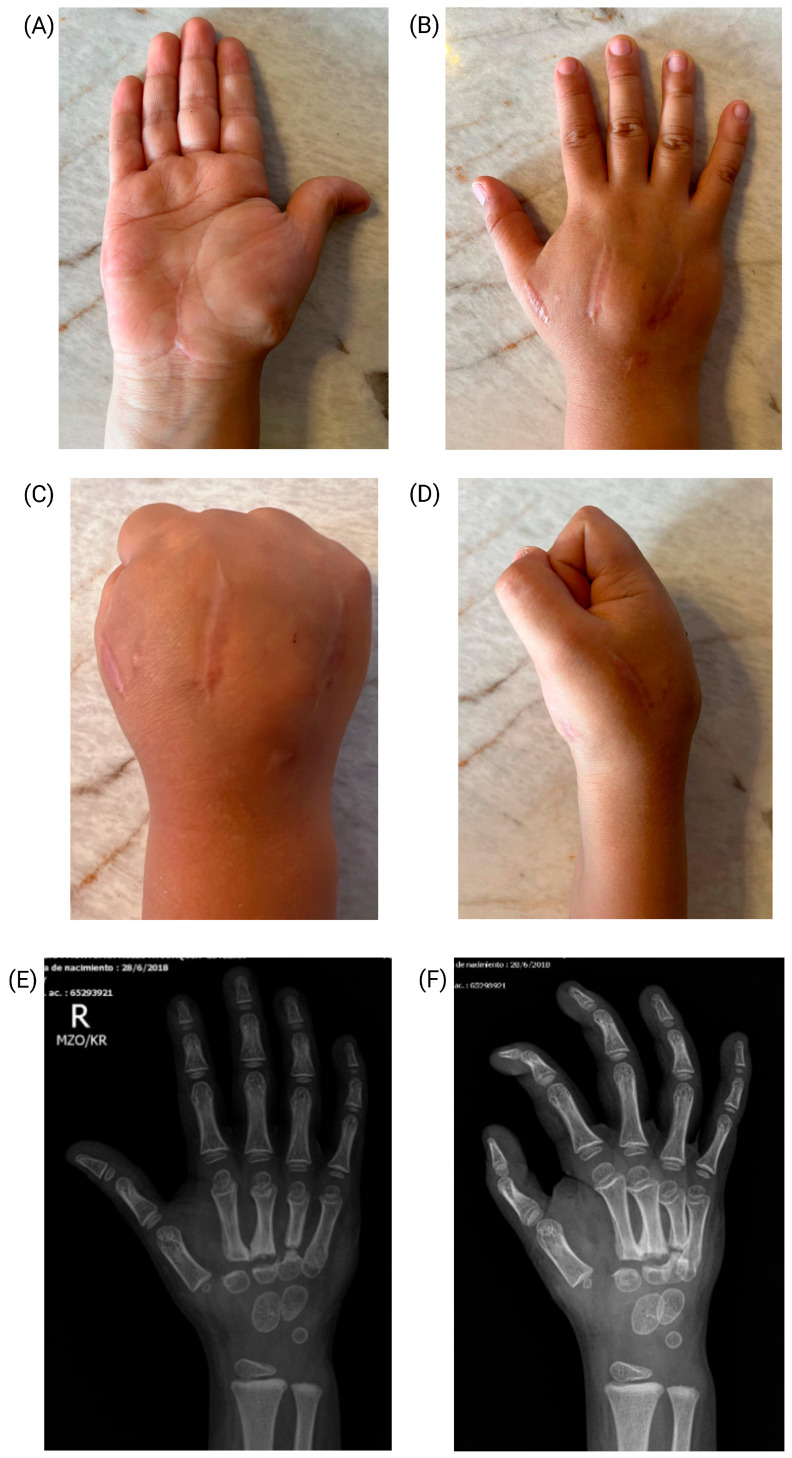
Functional recovery of the hand. (**A**) Palmar extension of the hand. (**B**) Dorsal extension of the hand. (**C**) Hand flexion in dorsal view. (**D**) Hand flexion in lateral view. (**E**) Anteroposterior radiograph of the hand showing callus formation and bone remodeling. (**F**) Oblique radiograph of the hand showing callus formation and bone remodeling.

At the three-month follow-up, the patient exhibited a complete functional recovery of the hand (Figure 4). Physical examination revealed an abduction range of 0° to 70° in the first digit, an interphalangeal joint flexion from 0° to 80°, and an interphalangeal extension from 0° to 20°. At the metacarpophalangeal level, mobility ranged from 0° to 45°. The second, third, fourth, and fifth fingers demonstrated a flexion from 0° to 90° and an extension from 0° to 45° at the metacarpophalangeal joints, with a proximal interphalangeal joint flexion ranging from 0° to 100°.

## 3. Discussion

We present the case of a 5-year-old pediatric patient who suffered a severe crush trauma to the right hand, resulting in ACS necessitating emergency surgical intervention.

While classic ACS symptoms in adults include pain, inflammation, paralysis, a diminished pulse, and neurological deficits, pediatric presentations are often more nonspecific [20]. Studies suggest that pain and swelling are the most common symptoms in pediatric upper extremity ACS, whereas pulselessness is less frequent [9]. In this case, the patient exhibited intense pain, deformity, edema, limited hand mobility, and delayed capillary refill, highlighting the diagnostic challenges of ACS in children. This case emphasizes that the classic adult “5 P’s” (pain, pallor, paresthesia, paralysis, and pulselessness) are not consistently reliable for early ACS detection in pediatric patients. Instead, clinicians should consider additional signs, such as those observed in our patient, to guide diagnosis. In addition, while hand ACS in adults is often caused by crush injuries, fractures, snake or insect bites, and burns, in children, it may also result from fractures (especially open), crush injuries, burns, vascular compromise, and, rarely, infections [9,21,22,23].

Regarding intracompartmental pressure, it is important to recognize that, in pediatric patients, particularly those with upper extremity fractures, compartment pressure measurements may not always be reliable. Previous research has shown that pediatric tissues can tolerate higher compartment pressures than those of adults, raising concerns about the accuracy of pressure-based diagnostic thresholds in this population [24]. These findings highlight the limitations of intracompartmental pressure measurements, especially in upper extremity cases, and reinforce the critical role of clinical suspicion and symptom recognition in the timely diagnosis and management of ACS in children [25].

ACS is less common in pediatric patients compared to adults, with upper extremity involvement being even rarer [7,10,26]. Trauma is identified as the primary etiology of upper extremity ACS in children, consistent with our patient’s case [11,20,27]. The literature on ACS management in pediatrics remains limited, with guidelines often extrapolated from adult studies, given their greater prevalence and research focus [28].

Several studies in the pediatric population emphasize the critical role of early surgical intervention, ideally within six hours, in preventing long-term complications [10,29]. In our case, surgery was performed within two hours, which likely contributed to the favorable long-term outcome. However, it is important to acknowledge that delayed surgical intervention may still yield positive results in this population. A systematic review and meta-analysis by Lin J. and Samora J. reported a mean time of 25.4 h to fasciotomy in 233 pediatric patients with compartment syndrome, with 85% achieving complete recovery—predominantly in the lower leg (60%) and forearm (27%) [30]. These findings suggest that, while early intervention is optimal, delayed surgery beyond 24 h can still be effective, reinforcing the need for individualized patient assessments when determining surgical timing.

Additionally, recent findings by Rademacher et al. (2020) indicate that delayed primary closure was successful in 77% of pediatric cases, a phenomenon which may be attributed to the greater wound elasticity observed in children compared to adults [18,31]. The positive evolution of our case underscores the crucial role of prompt identification and surgical intervention. Early decompression significantly reduces the risk of adverse outcomes, emphasizing that timely action is paramount regardless of whether the patient is a child or an adult.

In the management of compartment syndrome, decompression therapy options such as delayed primary closure and negative pressure wound therapy (NPWT), including the vacuum-assisted closure (VAC) technique, are widely recognized as being effective. While the VAC technique is a safe and reliable method for facilitating wound closure after fasciotomies, delayed primary closure offers comparable efficacy with the advantages of being faster to achieve a definitive closure and more cost-effective [32]. For our patient, we chose delayed primary closure due to its proven effectiveness and greater accessibility in resource-limited settings like ours [18].

Our patient’s management included multiple decompressive fasciotomies of the affected compartments of the hand, both dorsal and palmar, to alleviate pressure in key areas such as the interosseous, adductor, and thenar/hypothenar muscles. A central incision was performed to decompress the median nerve. While previous research indicates that success rates are not significantly influenced by the number of incisions [18], our approach highlights the importance of individualized management tailored to the severity of ACS and specific anatomical considerations, particularly in pediatric patients.

The fracture treatment was guided by the principles of early appropriate care, prioritizing the use of Kirschner wires (K-wires) to reduce complications associated with alternative internal fixation techniques [33]. K-wires were specifically selected to avoid the secondary trauma commonly associated with the second-strike phenomenon seen with rigid fixation methods, such as bone plates. Their use facilitated a successful closed reduction of the complex carpometacarpal dislocation, achieving the primary treatment goal while minimizing additional soft tissue damage.

Postoperatively, the patient underwent surgical wound cleaning every 72 h to monitor healing, showing optimal progress with reduced edema and improved finger mobility, without signs of infection. Early initiation of physical therapy played a crucial role in restoring finger mobility, acknowledging that complete recovery may require extended therapy periods.

## 4. Conclusions

Acute compartment syndrome in pediatric patients involving the upper extremities is rare and poses challenges in diagnosis and management. Despite the lack of specific guidelines for pediatric cases, this case contributes to the literature on ACS in children. It underscores the importance of early surgical decompression, the favorable outcomes observed with delayed primary closure of fasciotomies, and the need for individualized assessments to determine optimal treatment strategies within the surgical context.

## Data Availability

Due to the nature of this case report, the data supporting the findings cannot be shared publicly in order to protect patient confidentiality. However, further details may be made available by the corresponding author upon reasonable request.

## Abbreviations

The following abbreviations are used in this manuscript:

ACS	Acute compartment syndrome
VAC	Vacuum-assisted closure
NPWT	Negative pressure wound therapy
K-wires	Kirschner wires
